# Adipose-derived stem cells modified by TWIST1 silencing accelerates rat sciatic nerve repair and functional recovery

**DOI:** 10.1007/s13577-024-01087-6

**Published:** 2024-06-21

**Authors:** Bo Chen, Leining Wang, Xiaogui Pan, Shuai Jiang, Yihe Hu

**Affiliations:** 1 Department of Orthopedics, The First Affiliated Hospital, College of Medicine, Zhejiang University, No. 79 Qingchun Road, Hangzhou, 310003 Zhejiang China; 2 Department of Surgery of Hand and Foot, Beilun People’s Hospital, Ningbo, 315800 Zhejiang China

**Keywords:** Adipose-derived stem cells, Sciatic nerve, TWIST1, Neurotrophic factor, Cell therapy

## Abstract

The regeneration of peripheral nerves after injury is often slow and impaired, which may be associated with weakened and denervated muscles subsequently leading to atrophy. Adipose-derived stem cells (ADSCs) are often regarded as cell-based therapeutic candidate due to their regenerative potential. The study aims to assess the therapeutic efficacy of gene-modified ADSCs on sciatic nerve injury. We lentivirally transduced ADSCs with shRNA-TWIST1 and transplanted modified cells to rats undergoing sciatic nerve transection and repair. Results showed that TWIST1 knockdown accelerated functional recovery of rats with sciatic nerve injury as faster nerve conduction velocity and higher wire hang scores obtained by rats transplanted with TWIST1-silenced ADSCs than scramble ADSCs. Although the rats experienced degenerated axons and decreased myelin sheath thickness after sciatic nerve injury 8 weeks after operation, those transplanted with TWIST1-silenced ADSCs exhibited more signs of regenerated nerve fibers surrounded by newly formed myelin sheaths than those with scramble ADSCs. The rats transplanted with TWIST1-silenced ADSCs presented increased expressions of neurotrophic factors including neurotrophin-3 (NT-3), brain-derived neurotrophic factor (BDNF), nerve growth factor (NGF), and glial cell line-derived neurotrophic factor (GDNF) in the sciatic nerves than those with scramble ADSCs. These results suggest that genetically modifying TWIST1 in ADSCs could facilitate peripheral nerve repair after injury in a more efficient way than that with ADSCs alone.

**Supplementary Information:**

The online version contains supplementary material available at 10.1007/s13577-024-01087-6.

## Introduction

Peripheral nerve injuries remain a major clinical concern that often results from stretching or crushing trauma, penetrating trauma (gunshot wounds), motor vehicle accidents, and lacerations by sharp objects, leading to a significant decrease or even loss of motor and/or sensory function [1]. Peripheral nerves, unlike the central nervous system, have the ability to regenerate and remyelinate after injury, while this regeneration is limited in many ways such as loss of nerve segments and thus fails to achieve full restoration of function [2]. End-to-end neurorrhaphy remains the standard surgical technique for complete nerve transection but evidence supporting its use is limited to a shorter nerve gap (less than 1 cm) [3]. In cases of larger nerve gap, autologous nerve graft transplantation is the gold standard of treatment, but it is often limited by donor site morbidity [4]. The management of peripheral nerve injury to obtain satisfactory outcomes continues to be a major clinical challenge, and future attempts of care may focus on novel biologics and pharmacologic therapy to facilitate nerve regeneration and functional recovery [5].

The development of stem cell therapy involving versatile stem cells, such as bone marrow stem cells, adipose-derived stem cells, embryonic stem cells, and human umbilical cord stem cells, has brought a new perspective to bolster endogenous regenerative mechanisms or bioengineering new nerves [6, 7]. Bone marrow-derived mesenchymal stem cells (MSCs) have been demonstrated as an alternative to facilitate the healing of damaged peripheral nerves and restore functional recovery [8]. However, the procedures for harvesting BMSCs being invasive and painful with a low cellular yield create a critical need for identification of other sources of MSCs in transplantation therapy [9]. Adipose-derived stem cells (ADSCs) are believed to be an attractive candidate for regenerative medicine in treating peripheral nerve injury due to the ability of autologous transplantation concomitant with easy harvesting procedures [10]. The regenerative treatment modalities for ADSCs in treating peripheral nerve injury include differentiation into Schwann cells, promotion of myelination growth, secretion of neurotrophic factors to promote peripheral nerve growth, and combination of biomaterial scaffolds [11].

Various strategies have developed to improve efficacy of MSC-based therapy, among which the strategy using genetically modified MSCs that can improve their survival and tissue reconstructing abilities may represent the next stage of regenerative therapy [12]. TWIST1, an important bHLH transcription factor, is abundantly and selectively expressed in the adult adipose tissue and recognized as a potent regulator of adipose tissue remodeling and inflammation [13]. Numerous regulatory pathways dominate stem/progenitor and paracrine activities of MSCs seem to be linked mechanistically in a TWIST1-dependent manner [14]. Recent evidence shows genetically modified ADSCs with TWIST1 knockdown could increase osteogenic differentiation of ADSCs to enhance bone regeneration [15]. TWIST1 was found to suppress the activity of RUNX2 and RUNX2 inactivation may impair Schwann cell migration into the nerve bridge following nerve transection injury and remyelination, and thus delaying peripheral nerve regeneration [16]. Previous evidence showed that the expression of TWIST1 was elevated in rats with chronic sciatic nerve injury and downregulation of TWIST1 could depress neuropathic pain progression by alleviating neuroinflammation [17]. Accordingly, we proposed an intriguing hypothesis that TWIST1 may be involved in ADSC differentiation into Schwann cells and promotion of myelination growth. In this study, we lentivirally transduced ADSCs with shRNA-TWIST1 and transplanted modified cells to rats undergoing sciatic nerve transection and repair in a bid to investigate whether genetically modified ADSCs with TWIST1 knockdown could promote nerve regeneration and functional recovery compared to naïve ADSCs.

## Materials and methods

### Animals

Experiments involving animals were performed in 30 adult Sprague Dawley rats with body weigh ranging from 230 to 270 g which were purchased from the Experimental Animal Center of Xi’an Jiaotong University, housed in standard cages with free access to fresh water and standardized food in a temperature-controlled room (20–22 °C), and maintained on a 12 h light–dark cycle.

### Isolation and characterization of ADSCs

The fat tissue (10 g) was collected from the inguinal areas dissected from Sprague Dawley rats (n = 6), rinsed with saline, and cut into 1 mm^3^ pieces under aseptic conditions. The pieces were digested in 0.1% collagenase at 37 °C for 1 h and placed into the DMEM/F12 (Thermo Fisher Scientific, San Jose, CA, USA) containing 10% fetal bovine serum (FBS) (Sigma, NY, USA). The cells were pelleted by centrifugation (600×*g*) for 10 min, resuspended in 160 mM ammonium chloride for 3 min, and filtered through a 70-μm nylon mesh, followed by resuspension again in the DMEM/F12 and culture in 25-cm^2^ flasks (Nunc, Roskilde, Denmark) at 37 °C under 5% CO_2_. Once the cultures reached 80% confluence, the cells were detached using 0.25% trypsin–EDTA (Gibco, USA) and rinsed with phosphate-buffered saline (PBS). The ADSCs in passages 3–5 were analyzed by fluorescence-activated cell sorting using a CytoFLEX (Beckman Coulter, USA) with CD29-PE and CD34-FITC antibodies (eBioscience, San Diego, CA, USA). The ADSCs were characterized as CD29 + /high and CD34−/low.

### Transduction of ADSCs with lentiviral (LV) vectors

The ADSCs were plated onto 12-well plates and transduced with either 12 ml of LV particles containing shRNA targeting TWIST1 (sc-38604-V; Santa Cruz Biotechnology, Santa Cruz, CA, USA) or LV particles containing scramble shRNA (sc-108080; Santa Cruz Biotechnology) each well in DMEM added with GlutaMAX plus 5 mg/ml polybrene for 6 h to achieve TWIST1 knockdown.

### ADSC differentiation into Schwann cells

The ADSCs were seeded in duplicate on 6-well plates and maintained in culture medium until reaching approximately 90% confluency. To induce Schwann cells, ADSCs were allowed to undergo a 24 h treatment with 1 mM β-mercaptoethanol, followed by a 72 h treatment with 35 ng/mL all-trans retinoic acid. Both substances were dissolved in growth medium, composed of 90% Minimum Essential Medium (α-MEM), 2 nM L-glutamine, and 10% fetal bovine serum. The cells were then differentiated for 14 days under specific differentiation conditions. The differentiation medium included growth medium supplemented with 5 ng/mL platelet-derived growth factor, 10 ng/mL basic fibroblast growth factor, 14 mM forskolin, and 192 ng/mL glial growth factor 2. The medium was changed every 2–3 days. After 14 days, the morphology of ADSCs was observed using an inverted microscope.

### Surgical procedures and application of ADSCs

Rats were randomly arranged into non-lesioned, non-transplanted, LV-scramble-ADSCs, and LV-sh-TWIST1-ADSCs groups. Usually, end-to-end neurorrhaphy was performed for a shorter nerve gap (less than 1 cm) after sciatic nerve repair [3]. As a previously reported protocol, a 5 mm gap defect was surgically created in the sciatic nerve for rats undergoing sciatic nerve transection and repair [18]. Briefly, the rats were anesthetized with isoflurane and placed in a right lateral position. A gluteal skin incision was made from the sciatic notch to a point proximal to the knee joint. The sciatic nerve from the sciatic notch to the point of bifurcation was exposed, sharply transected at 1 cm proximal to the trifurcation to avoid saw contusion, and subsequently reconnected with four 10/0 epineurial nylon sutures using microscopic visualization. The rats in the LV-scramble-ADSCs and LV-sh-TWIST1-ADSCs groups received a local injection of 5 × 10^4^ LV transduced ADSCs (reconstituted to a 5 μl volume) into the transected sciatic nerve stumps with a 10 L syringe. The rats in the non-transplanted groups received normal saline injection as same volume. In the non-lesioned group, rats underwent a sham operation followed by normal saline injection as same volume only as a reference for functional normality after nerve exposure. After operation, all rats were monitored for signs of infection or distress.

### Electrophysiological analysis

The electrophysiological analysis was performed to record nerve conduction velocity of rats at 8 weeks after the surgical procedure. The rats were anesthetized with pentobarbital and then the sciatic nerve of the operated side was re-exposed. The proximal and distal ends of the regenerating nerve trunk were respectively given the electrical stimuli. The nerve conduction velocity was recorded on the gastrocnemius belly at the ipsilateral side using an electromyography recorder (CareFusion, CA, USA). The stimulating mode was set to the pulse mode (frequency = 1 Hz, duration = 1 ms, level = 39.2 mA).

### Wire hang tests

A 50 cm steel wire (2 mm in diameter) was stretched horizontally 40 cm above floor level and the rates were allowed to grasp the middle of the steel wire with their two forepaws for 30 s in three replications. The rats falling off were scored as 0, those hanging on the steel wire by two forepaws were scored as 1, those hanging on the steel wire by two forepaws but attempting to climb on the steel wire were scored as 2, those hanging on the steel wire by two forepaws plus one or both hind paws were scored as 3, those hanging on the steel wire by all four paws plus the tail wrapped around the wire were scored as 4, and those escaped to one of the platforms at each end of the wire were scored as 5.

### Histomorphological analysis

The 1.5 cm segments of the tibial and fibular portions of the sciatic nerve at its bifurcation were collected from the lesioned limbs of rats after 8 week would closure. The sciatic nerve segments were immersed in a 25 g/L glutaraldehyde solution, fixed with 10% formalin containing 1% glutaraldehyde and 1% sucrose overnight at 4 ℃ for 12 h, rinsed 3 times with PBS for 5 min each time, and colored with 10 g/L osmic acid at 37 ℃, followed by gradient dehydration and directional embedding. The sciatic nerve segments were thin-sectioned and stained with 1% toluidine blue, followed by microscopic visualization.

### Quantitative real-time PCR (qRT-PCR)

After extracting total RNA from collected sciatic nerve sections using the Trizol reagents (Invitrogen, Carlsbad, CA, USA) to synthesize cDNA using the PrimeScript RT Reagent kit (Takara, Dalian, China), expressions of TWIST1, NT-3, BDNF, NGF, and GDNF were quantified by the qRT-PCR (Table 1 lists the primer sequences) using the SYBR Master Mixture (Takara, Tokyo, Japan) and the LightCycler 480 II System (Roche Diagnostics, Indianapolis, IN, USA). Amplification of GAPDH served as a loading control.

**Table 1:** The primer sequences used for the qRT-PCR analysis

Target	Primer sequence	Length (bp)
TWIST1 (NM_053530.2)	Sense: 5′-TTTTAAAAGTGCGCCCCACG-3′	20
	Antisense: 5′-ACAGCCGCAGAGACCTAAAC-3′	20
NT-3 (NM_031767.1)	Sense: 5′-TTGCAGAAGCAGCTAGCCAT-3′	20
	Antisense: 5′-CCCTTTAGCACCAATCCGGT-3′	20
BDNF (NM_012513.4)	Sense: 5′-TGTGTCTATGCCTGGGGCTA-3′	20
	Antisense: 5′-AGCACTAGCTGCCTATTCCAA-3′	21
NGF (NM_001277055.1)	Sense: 5′-GAATTCGCCCCTGTGGAAGA-3′	20
	Antisense: 5′-AGTGCCCCGGTTTGAATGAA-3′	20
GDNF (NM_019139.2)	Sense: 5′-CAGAATGCACGTTAAGCCTGG-3′	21
	Antisense: 5′-AACTGGGGACTTGCAAGGAG-3′	20
GAPDH (NM_017008.4)	Sense: 5′-TTAGCACCCCTGGCCAAGG-3′	19
	Antisense: 5′-CTTACTCCTTGGAGGCCATG-3′	20

### Immunoblotting analysis

The collected sciatic nerve sections reacted in the RIPA lysis buffer, followed by sodium dodecyl sulfate–polyacrylamide gel electrophoresis separation and transfer onto polyvinylidene fluoride membrane. Immunoblots were visualized after incubation with primary antibodies: mouse monoclonal anti-rat TWIST1 antibody (ab50887, Abcam, Cambridge, UK), rabbit monoclonal anti-rat NT-3 antibody (ab263864, Abcam), rabbit monoclonal anti-rat BDNF antibody (ab108319, Abcam), rabbit monoclonal anti-rat NGF antibody (MA5-32067, eBioscience), and mouse monoclonal anti-rat GDNF antibody (sc-13147, Santa Cruz Biotechnology, CA, USA), followed by incubation with secondary antibodies. GAPDH was used as a loading control. Densitometry analysis of immunoblots was carried out with the aid of the ImageJ software program (NIH, Bethesda, MA). The density of each immunoblot was normalized to that of GAPDH.

### Statistical analysis

The outcomes yielded from at least three independent samples and involving at least three individual experiments were summarized using mean ± standard deviation. All statistical analyses including unpaired t-test, one-way analysis of variance (ANOVA), and repeated measures ANOVA were performed with the aid of GraphPad Prism version 8.0 (GraphPad Software, La Jolla, CA, USA) for Windows. *P* < 0.05 was indicative of significant differences.

## Results

### *Characterization of ADSCs and their d*ifferentiation into Schwann cells

**Fig. 1:**
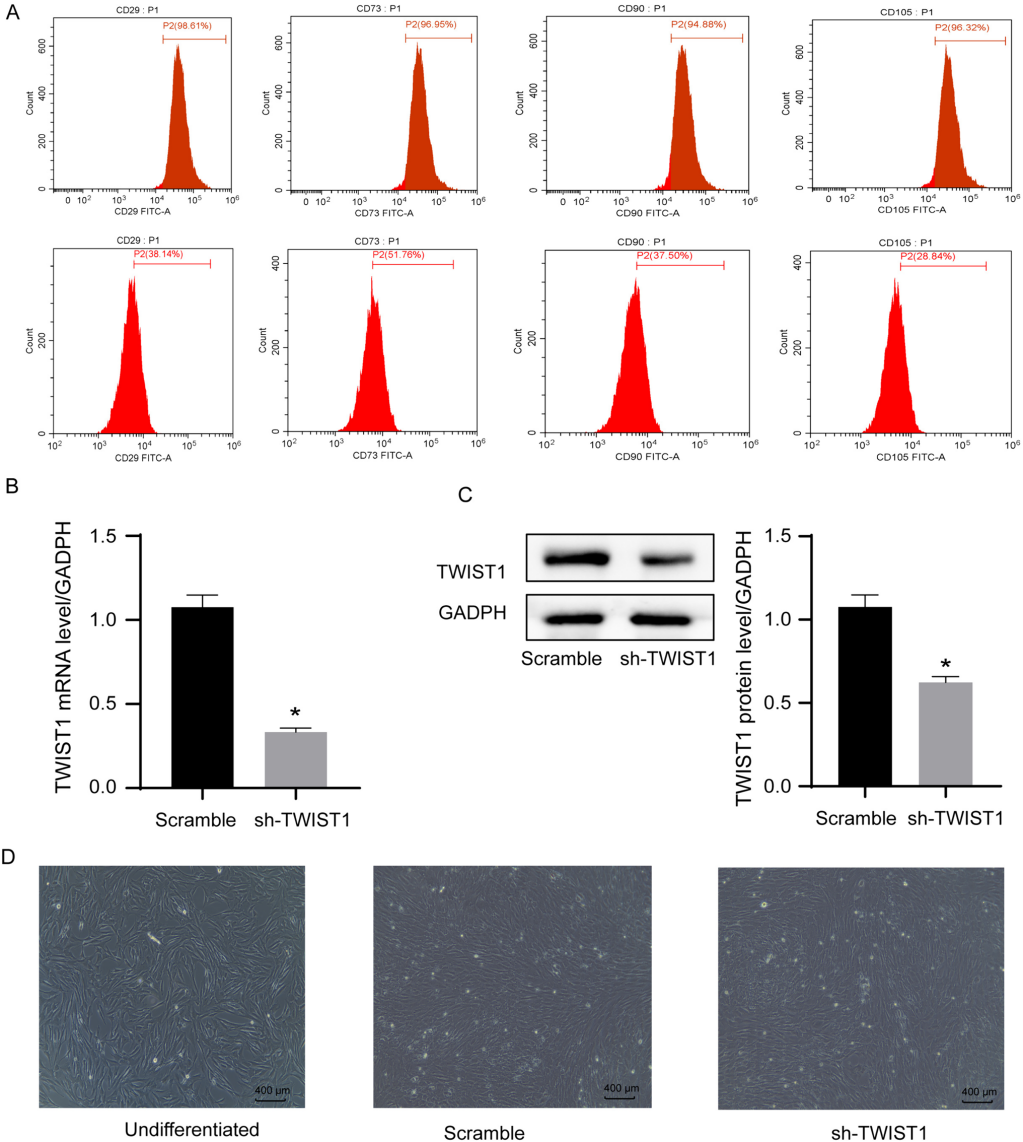
Characterization of ADSCs and their differentiation into Schwann cells. A, Phenotypic characterization of ADSCs by flow cytometric analysis. B, The qRT-PCR analysis of TWIST1 mRNA expression levels in ADSCs 2 weeks after LV transduction. C, Immunoblotting analysis of TWIST1 protein expression levels in ADSCs 2 weeks after LV transduction. *P < 0.05 compared to the scramble siRNA by unpaired t tests. D, Representative inverted microscope images of ADSCs under Schwann cell induction for 14 days. Without induction, ADSCs showed a mesh-like structure. After 14 days, cells adopted a spindle shape with reduced volume, fewer protrusions, and a spiral growth pattern, resembling Schwann cells. Notably, the sh-TWIST1 group exhibited more pronounced Schwann cell-like features than the scramble group

Flow cytometric analysis found the isolated and cultured cells were positive for CD29 (98.61%), CD73 (96.95%), CD90 (94.88%), and CD105 (96.32%) (Fig. 1A). The qRT-PCR (Fig. 1B) and the immunoblotting analysis (Fig. 1C) were carried out to assess TWIST1 mRNA and protein expression levels in ADSCs 2 weeks after LV transduction. A reduced TWIST1 expression was noted in ADSCs transducted with LV vectors carrying sh-TWIST1 compared to those transducted with LV vectors carrying scramble shRNA. Morphologically, ADSCs undergoing Schwann cell induction displayed distinctive changes, with ADSCs transducted with LV vectors carrying sh-TWIST1 showing more pronounced Schwann cell-like features than those transducted with LV vectors carrying scramble shRNA (Fig. 1D).

### *ADSCs with TWIST1 knockdown accelerated* functional recovery of rats with sciatic nerve injury

**Fig. 2:**
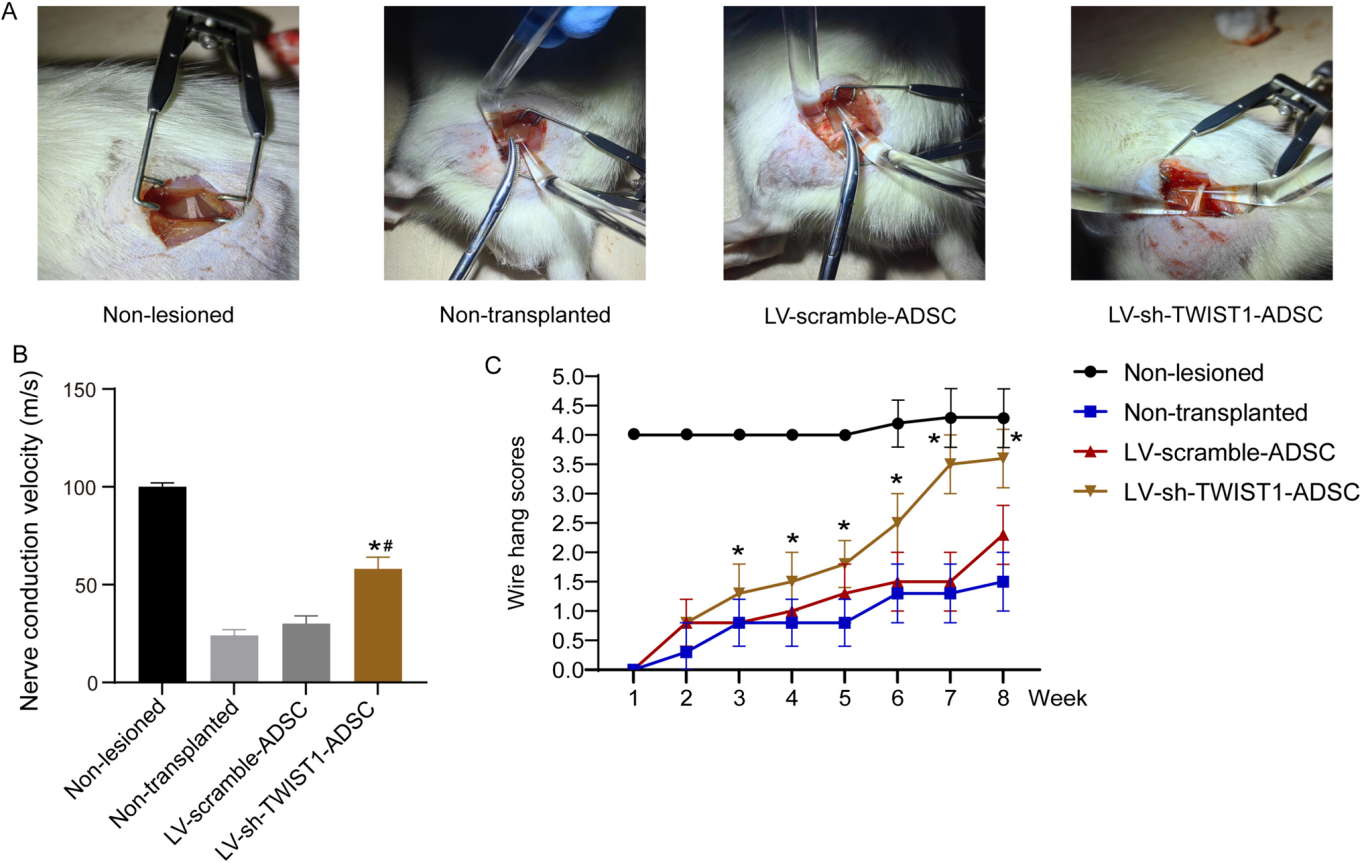
The functional recovery of rats with sciatic nerve injury after injections of ADSCs. A, Rat models of sciatic nerve injury. B, Quantification of nerve conduction velocity in the indicated groups at week 8 after transplantation of ADSCs; *P < 0.05 compared to the non-transplanted group and #*P* < 0.05 compared to the LV-scramble-ADSC group by one-way ANOVA followed by Tukey’s multiple comparisons test. C, The wire hang scores of rats with sciatic nerve injury at indicated time points with or without transplantation of ADSCs; *P < 0.05 by repeated measures ANOVA

To assess the effect of ADSCs on sciatic nerve function, the rats underwent surgical procedures involving sciatic nerve transection and repair, and then received injections of ADSCs (Fig. 2A). We next examined the nerve conduction velocity of the regenerated sciatic nerves in rats 8 weeks after the repair. The nerve conduction velocity of the rats transplanted LV-sh-TWIST1-ADSC was significantly faster than that of those transplanted LV-scramble-ADSC and non-transplanted rats (*P* < 0.05) (Fig. 2B). The wire hang score curves showed that rats transplanted LV-sh-TWIST1-ADSC exhibited higher wire hang scores indicating better functional recovery from 3 weeks after operation compared to those transplanted LV-scramble-ADSC and non-transplanted rats (Fig. 2C). No significant difference was observed between rats transplanted LV-scramble-ADSC and non-transplanted rats.

### *ADSCs with TWIST1 knockdown promoted sciatic nerve repair in rats* with sciatic nerve injury

**Fig. 3:**
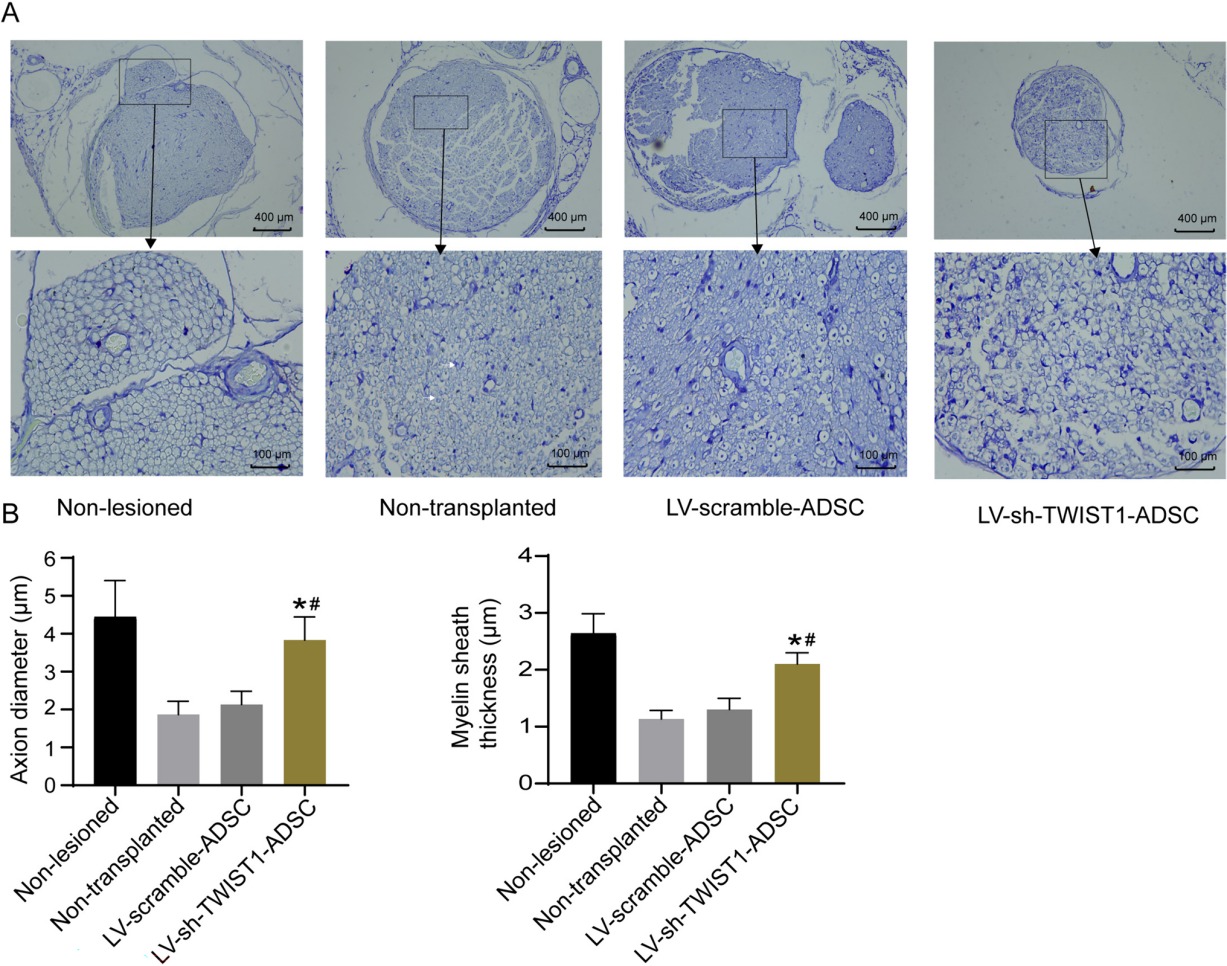
The sciatic nerves of rats in non-lesioned, non-transplanted, LV-scramble-ADSC, and LV-sh-TWIST1-ADSC groups 8 weeks after transplantation of ADSCs were collected and subjected to histomorphological analysis. A, The sections of rat sciatic nerve stained with toluidine blue; the non-lesioned rats showed homogeneously distributed fibers, uniform myelin sheath, and normal axon diameter; the non-transplanted rats showed clear signs of nerve fiber degeneration with presence of myelin degradation, indicated by white arrows; the LV-scramble-ADSC rats showed some signs of regenerated nerve fibers but presence of degenerated axons and decreased myelin sheath thickness; the LV-sh-TWIST1-ADSC rats showed clear signs of regenerated nerve fibers surrounded by newly formed myelin sheaths. B, Morphometric analysis of axion diameter and myelin sheath thickness. *P < 0.05 compared to the non-transplanted group and #*P* < 0.05 compared to the LV-scramble-ADSC group by one-way ANOVA followed by Tukey’s multiple comparisons test

The sciatic nerves of rats in non-lesioned, non-transplanted, LV-scramble-ADSC, and LV-sh-TWIST1-ADSC groups 8 weeks after operation were collected and subjected to histomorphological analysis (Fig. 3A). The non-lesioned group showed homogeneously distributed fibers, clear edges, normal number of myelinated nerve fibers, uniform myelin sheath, and normal axon diameter. The non-transplanted group showed disorganized nerve fibers crowded with each other with presence of thin myelin sheath and degenerated axons. The LV-scramble-ADSC group showed some signs of regenerated nerve fibers but presence of degenerated axons and decreased myelin sheath thickness. The LV-sh-TWIST1-ADSC group showed clear signs of regenerated nerve fibers surrounded by newly formed myelin sheaths with a clear morphology, an increased number of myelinated nerve fibers with an increased thickness in myelin sheath and axons. Although the non-transplanted, LV-scramble-ADSC, and LV-sh-TWIST1-ADSC groups all presented a certain number of abnormal myelinated nerve fibers and a certain degree of axon extension, the non-transplanted and the LV-scramble-ADSC groups exhibited more abnormal myelinated nerve fibers than LV-sh-TWIST1-ADSC group. No significant difference was observed between the non-transplanted group and LV-scramble-ADSC group. Morphometric analysis (Fig. 3B) demonstrated significantly greater axion diameter and myelin sheath thickness in the LV-sh-TWIST1-ADSC group compared to the other three groups (Fig. 3B, *P* < 0.05).

### *ADSCs with TWIST1 knockdown was associated with increased expressions of neurotrophic factors in rats* with sciatic nerve injury

**Fig. 4:**
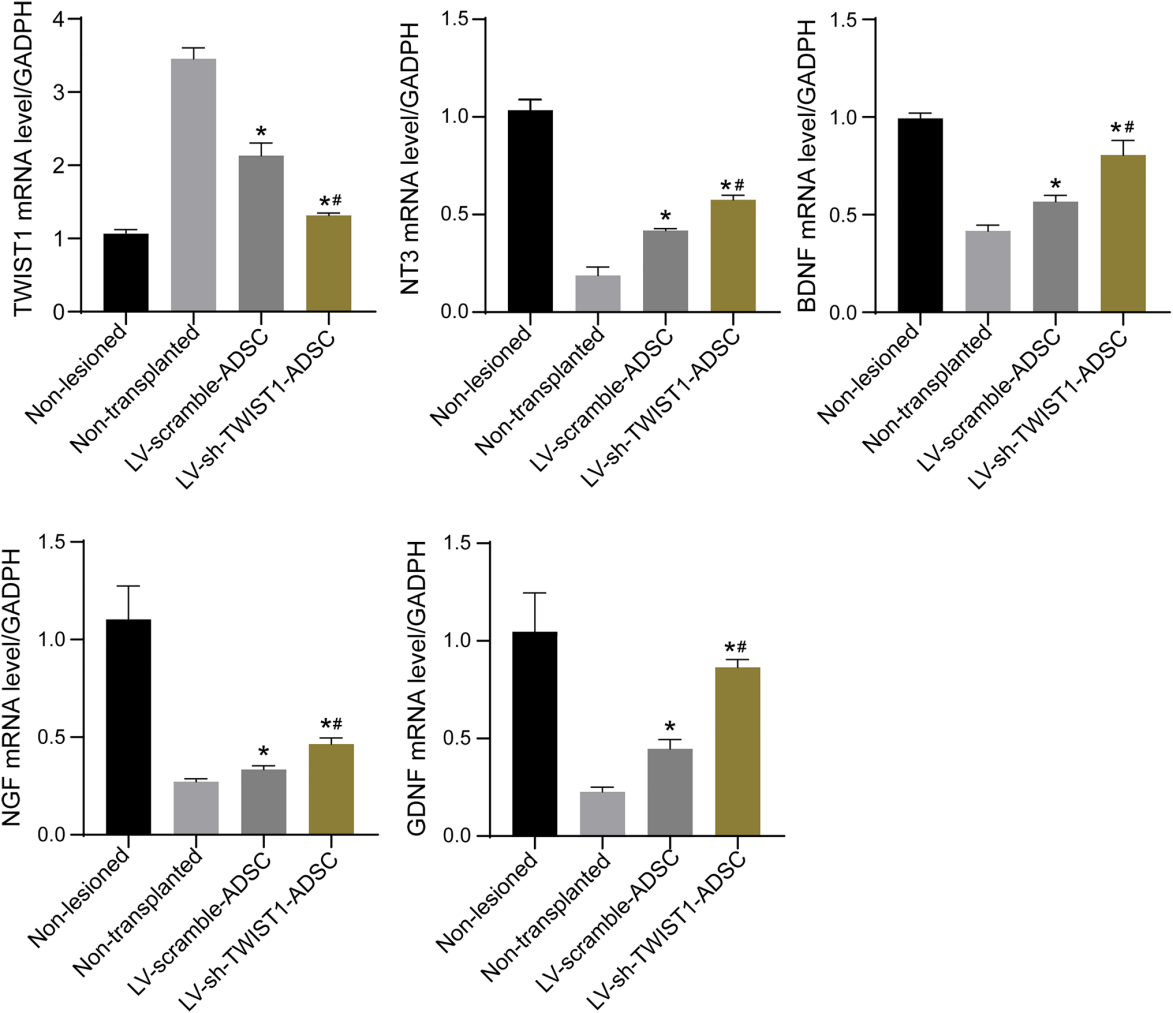
The mRNA expressions of TWIST1, NT-3, BDNF, NGF, and GDNF in the sciatic nerves of rats after sciatic repair in non-lesioned, non-transplanted, LV-scramble-ADSC, and LV-sh-TWIST1-ADSC groups 8 weeks after transplantation of ADSCs were determined by the qRT-PCR analysis. *P < 0.05 compared to the non-transplanted group and #*P* < 0.05 compared to the LV-scramble-ADSC group by one-way ANOVA followed by Tukey’s multiple comparisons test

**Fig. 5:**
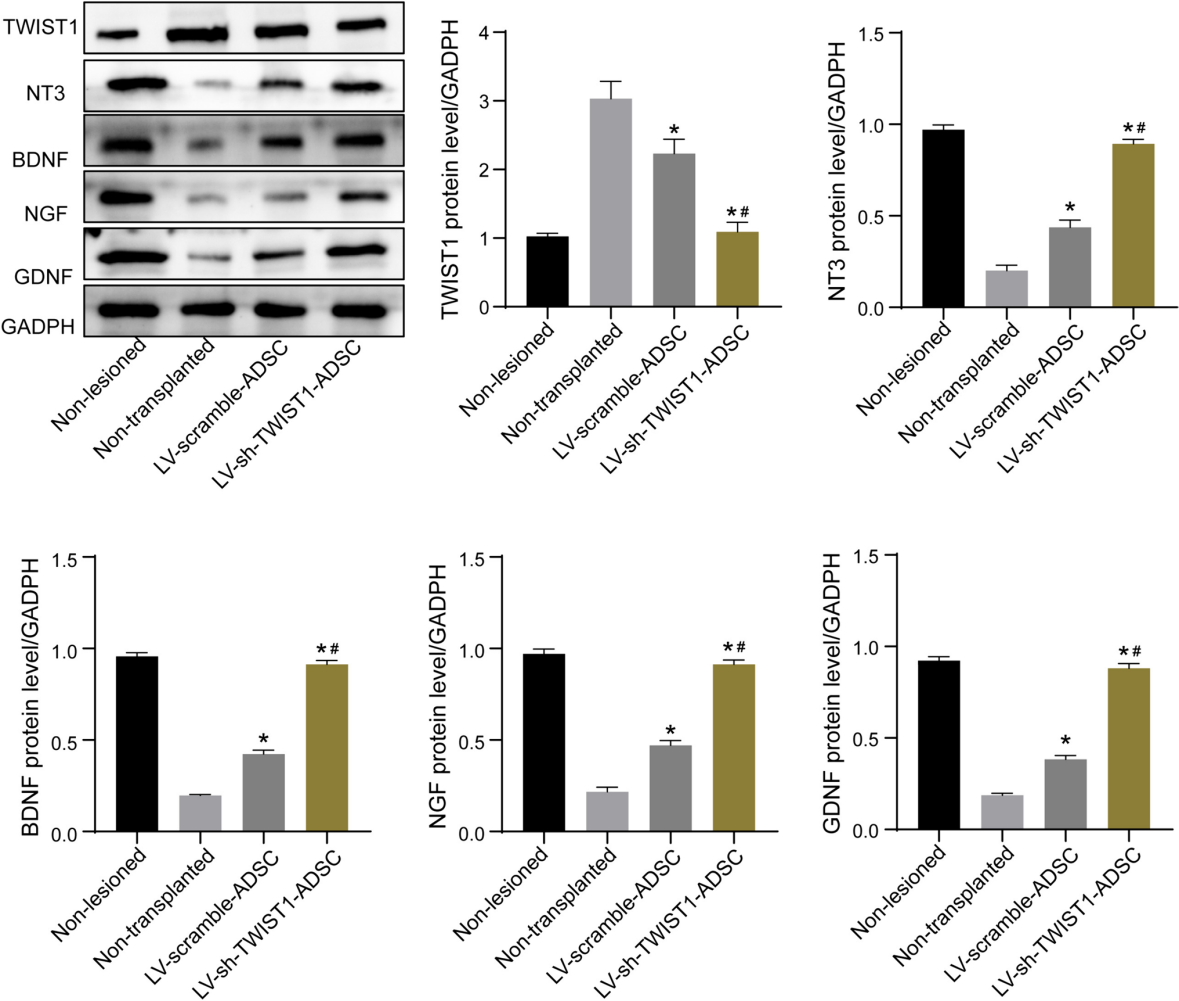
The protein expressions of TWIST1, NT-3, BDNF, NGF, and GDNF in the sciatic nerves of rats after sciatic repair in non-lesioned, non-transplanted, LV-scramble-ADSC, and LV-sh-TWIST1-ADSC groups 8 weeks after transplantation of ADSCs were determined by the immunoblotting analysis. *P < 0.05 compared to the non-transplanted group and #*P* < 0.05 compared to the LV-scramble-ADSC group by one-way ANOVA followed by Tukey’s multiple comparisons test

Neurotrophin-3 (NT-3), brain-derived neurotrophic factor (BDNF), glial cell derived neurotrophic factor (GDNF), and nerve growth factor (NGF), four important neurotrophic factors supporting Schwann cell survival and differentiation and stimulating axon regeneration and myelination, have proven to be critical for promoting peripheral nerve regeneration [19–22]. To determine the therapeutic potential of ADSCs with TWIST1 knockdown for the process of peripheral nerve regeneration, we examined the expressions of NT-3, BDNF, NGF, and GDNF in the sciatic nerves of rats after sciatic repair by the qRT-PCR (Fig. 4) and immunoblotting analysis (Fig. 5). At 8 weeks following the application of ADSCs, the expressions of TWIST1 mRNA and protein in the sciatic nerves of rats in the LV-sh-TWIST1-ADSC group were remarkably reduced compared to the LV-scramble-ADSC group and non-transplanted group. The expressions of NT-3, BDNF, NGF, and GDNF mRNA and protein in the sciatic nerves of rats in the LV-sh-TWIST1-ADSC group were remarkably increased compared to the LV-scramble-ADSC group and non-transplanted group. No significant differences with regard to expressions of TWIST1, NT-3, BDNF, NGF, and GDNF mRNA and protein were observed between the non-transplanted group and LV-scramble-ADSC group.

## Discussion

**Fig. 6:**
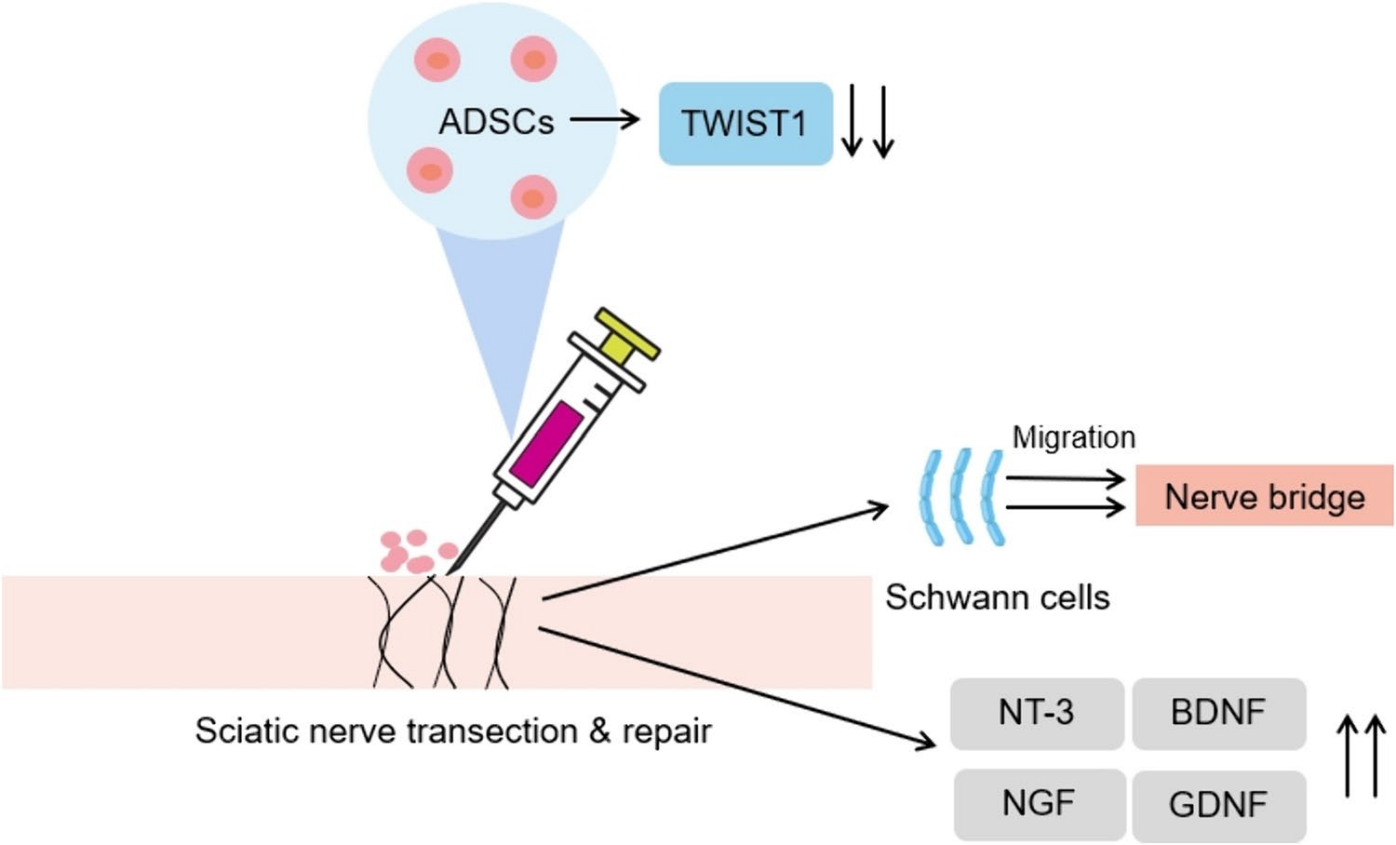
Schematic summary of the therapeutic benefit of ADSCs for rats undergoing sciatic nerve injury

Previous evidence supports limited power of naïve BMSCs and ADSCs to offer satisfactory outcomes for peripheral nerve regeneration [23]. In an attempt to optimize MSC-based therapy for peripheral nerve repair, we lentivirally transduced ADSCs with shRNA-TWIST1 to achieve WIST1 silencing and explored the nerve regeneration and functional recovery of rats after sciatic nerve transection and repair followed by topical application of genetically-manipulated ADSCs. Our data show that TWIST1 silencing could improve the efficacy of ADSCs to facilitate nerve regeneration and functional recovery of rats with peripheral nerve injury (Fig. 6).

A topical form of cell therapy using different multipotent stem cells including ADSCs has been reported previously for regeneration medicine [24, 25]. New knowledge about the way in which signals control peripheral nerve regeneration has promoted the topical application of multipotent stem cells and bioactive molecules to damaged nerves [26]. TWIST1 was previously found to be increased in rats with chronic sciatic nerve injury and downregulation of TWIST1 could depress neuropathic pain progression by alleviating neuroinflammation [17]. In this study, we demonstrated TWIST1 inhibition in ADSCs was beneficial for peripheral nerve regeneration. In vitro and in vivo osteogenic differentiation of ADSCs was enhanced by TWIST1 silencing contributing to TAZ upregulation [15]. TAZ is required for Schwann cells redifferentiation into myelinating Schwann cell during peripheral nerve regeneration [27]. TWIST1-null bone marrow-derived matrix-producing cells were characterized by high levels of the T cell chemoattractant CXCL12, and CXCL12 peptide promoted functional neuronal connections in rats after spinal cord injury [28, 29]. TWIST1 is known as a functional antagonist of RUNX2 whose expression may be associated with outgrowth of neurites and Schwann cell differentiation and myelinization after sciatic nerve crush [30–32]. TWIST1 silencing may liberate RUNX2 expression, its functional antagonist, thus leading to RUNX2 activation, which subsequently promotes Schwann cell migration into the nerve bridge following nerve transection injury and remyelination [16, 33]. Earlier work has described the importance of TWIST1 in the pathogenesis of malignant peripheral nerve sheath tumors [34], indicating the potential role of TWIST1 in regulating peripheral nerves. Given that our study is the first study reporting the beneficial effects of TWIST1 inhibition in ADSCs for peripheral nerve regeneration, further investigations are required to decipher the underlying mechanism of TWIST1 silencing for nerve regeneration.

ADSCs have also been shown to be able to differentiate to Schwann-like cells that can contribute to peripheral nerve regeneration [35]. The secretion of neurotrophic factors, such as NT-3, BDNF, NGF, and GDNF, can accelerate and trigger axonal regrowth, which may contribute to accelerated nerve for the treatment of peripheral nerve regeneration [36]. ADSCs secrete functional neurotrophic and angiogenic factors including BDNF, GDNF, vascular endothelial growth factor-A (VEGF-A), and angiopoietin-1, creating a more desirable microenvironment for nerve regeneration [37]. Synergistic overexpression of BDNF and NT‑3 has the ability to promote the neuronal differentiation of ADSCs, which was in line with our results that increased expressions of NT-3 and BDNF after TWIST1 silencing to enhance the sciatic nerve repair conferred by ADSCs [38]. When TWIST1 expression was inhibited in ADSCs, NT-3, BDNF, NGF, and GDNF were enhanced thus stimulates neurotrophic properties of ADSCs. In a previous study, human platelet lysate-cultured ADSCs resulted in enhanced neurotrophic properties showing higher gene expression of NGF compared to Schwann cells differentiated from ADSCs [39]. TWIST1 knockdown in mutant huntingtin-expressing primary cortical neurons reversed robust gene expression changes related to neuronal function and, importantly, reversed mutant huntingtin-induced DNA hypermethylation at the BDNF regulatory region and reactivate the expression of BDNF [40]. No previous reports showing the regulation of TWIST1 on NT-3 and GDNF expressions promote us to elucidate exact mechanism of TWIST 1 mediating gene induction in further studies. For example, further studies are necessary to perform immunohistochemical analysis of NT-3, BDNF, NGF, and GDNF in the sciatic nerves of rats after sciatic repair to confirm the therapeutic potential of ADSCs with TWIST1 knockdown for the process of peripheral nerve regeneration.

Our data suggest that TWIST1 RNA interference provides additional benefits of nerve regeneration and functional recovery to enhance the efficacy of topical application of ADSCs for peripheral nerve injury treatment, improving their neurotrophic properties and concurrently maintaining them in a neuroinduced state while preserving their stem properties. This is particularly important when TWIST1-silenced ASCs are considered as potential candidate for injective treatments in the context of regenerative therapy.

### Supplementary Information

Below is the link to the electronic supplementary material.

Supplementary file1 (DOCX 515 KB)

## Data Availability

The data used for the study are available in the present study.

## Abbreviations

ADSCs: Adipose-derived stem cells
NT-3: Neurotrophin-3
BDNF: Brain-derived neurotrophic factor
NGF: Nerve growth factor
GDNF: Glial cell line-derived neurotrophic factor
MSCs: Mesenchymal stem cells
FBS: Fetal bovine serum
PBS: Phosphate-buffered saline
LV: Lentiviral
MEM: Minimum essential medium
qRT-PCR: Quantitative real-time PCR
ANOVA: One-way analysis of variance
