# Supplementary figures and images for: Adipose-derived stem cells modified by TWIST1 silencing accelerates rat sciatic nerve repair and functional recovery
Source: Hum Cell. 2024 Jun 21;37(5):1394–404. doi: 10.1007/s13577-024-01087-6 (PMC11341607; doi:10.1007/s13577-024-01087-6)

**Figure 1**

**TWIST1:**

**

**

**GAPDH1:**

**

**

**Figure 2**

**TWIST1:**

**

**

**NT-3:**

**

**

**BDNF:**

**
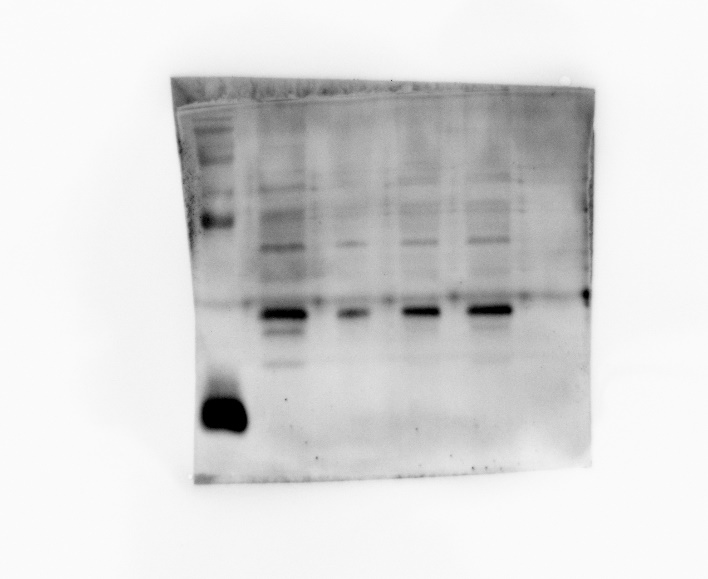
**

**NGF:**

**

**

**GDNF:**

**

**

Supplement: Supplementary file 1 — Supplementary file1 (DOCX 515 KB) [file 13577_2024_1087_MOESM1_ESM.docx]
